# Supplementary material for: Gender disparities in Italian academic medicine: A cross-sectional study of clinicians in the 2024 stanford top 2% scientists database
Source: PLoS One. 2026 Apr 24;21(4):e0347836. doi: 10.1371/journal.pone.0347836 (PMC13108791; doi:10.1371/journal.pone.0347836)
Supplement: S2 Text — (DOCX) [file pone.0347836.s002.docx]

**Supplementary material**

***eTable 1:Distribution of gender per academic field***

| **Field** | **Women n (%)** | **Men n (%)** |
| --- | --- | --- |
| Allergy | 3 (15.8%) | 16 (84.2%) |
| Anatomy & Morphology | 4 (44.4%) | 5 (55.6%) |
| Anesthesiology | 10 (23.8%) | 32 (76.2%) |
| Arthritis & Rheumatology | 15 (23.1%) | 50 (76.9%) |
| Cardiovascular System & Hematology | 55 (16.0%) | 289 (84.0%) |
| Dentistry | 29 (18.4%) | 129 (81.6%) |
| Dermatology & Venereal Diseases | 26 (33.3%) | 52 (66.7%) |
| Emergency & Critical Care Medicine | 3 (7.7%) | 36 (92.3%) |
| Endocrinology & Metabolism | 28 (25.2%) | 83 (74.8%) |
| Epidemiology | 4 (57.1%) | 3 (42.9%) |
| Gastroenterology & Hepatology | 38 (20.8%) | 145 (79.2%) |
| General & Internal Medicine | 37 (35.2%) | 68 (64.8%) |
| General Clinical Medicine | 26 (42.6%) | 35 (57.4%) |
| Genetics & Heredity | 5 (19.2%) | 21 (80.8%) |
| Geriatrics | 3 (17.6%) | 14 (82.4%) |
| Gerontology | 4 (50.0%) | 4 (50.0%) |
| Health Policy & Services | 2 (50.0%) | 2 (50.0%) |
| History of Science, Technology & Medicine | 1 (100.0%) | 0 (0.0%) |
| Immunology | 48 (29.6%) | 114 (70.4%) |
| Legal & Forensic Medicine | 15 (41.7%) | 21 (58.3%) |
| Microbiology | 61 (37.2%) | 103 (62.8%) |
| Mycology & Parasitology | 5 (29.4%) | 12 (70.6%) |
| Neurology & Neurosurgery | 77 (24.9%) | 232 (75.1%) |
| Nuclear Medicine & Medical Imaging | 24 (21.4%) | 88 (78.6%) |
| Nutrition & Dietetics | 19 (43.2%) | 25 (56.8%) |
| Obstetrics & Reproductive Medicine | 34 (23.0%) | 114 (77.0%) |
| Oncology & Carcinogenesis | 110 (32.6%) | 227 (67.4%) |
| Ophthalmology & Optometry | 7 (10.9%) | 57 (89.1%) |
| Orthopedics | 7 (13.7%) | 44 (86.3%) |
| Otorhinolaryngology | 8 (12.7%) | 55 (87.3%) |
| Pathology | 9 (33.3%) | 18 (66.7%) |
| Pediatrics | 22 (29.3%) | 53 (70.7%) |
| Physiology | 1 (7.7%) | 12 (92.3%) |
| Psychiatry | 13 (26.5%) | 36 (73.5%) |
| Public Health | 2 (20.0%) | 8 (80.0%) |
| Rehabilitation | 3 (20.0%) | 12 (80.0%) |
| Respiratory System | 10 (17.9%) | 46 (82.1%) |
| Substance Abuse | 0 (0.0%) | 1 (100.0%) |
| Surgery | 10 (6.1%) | 153 (93.9%) |
| Toxicology | 24 (63.2%) | 14 (36.8%) |
| Tropical Medicine | 3 (30.0%) | 7 (70.0%) |
| Urology & Nephrology | 14 (11.6%) | 107 (88.4%) |
| Virology | 4 (16.7%) | 20 (83.3%) |

*n:number*

***eTable 2. Logistic Regression***

| Variable | OR (IC95%) | P-value |
| --- | --- | --- |
| **Academic affiliation** | 0.93 (0.77; 1.13) | 0.472 |
| **Year of first publication** | 1.02 (1.02; 1.03) | <0.001 |
| **Field** |  |  |
| *Anatomy & Morphology* | 3.59 (0.60; 24.62) | 0.168 |
| *Anesthesiology* | 1.30 (0.34; 6.45) | 0.719 |
| *Arthritis & Rheumatology* | 1.48 (0.42; 7.01) | 0.576 |
| *Cardiovascular System & Hematology* | 0.93 (0.29; 4.10) | 0.908 |
| *Dentistry* | 0.94 (0.29; 4.26) | 0.927 |
| *Dermatology & Venereal Diseases* | 2.28 (0.68; 10.49) | 0.224 |
| *Emergency & Critical Care Medicine* | 0.34 (0.06; 2.05) | 0.223 |
| *Endocrinology & Metabolism* | 1.81 (0.55; 8.22) | 0.377 |
| *Epidemiology* | 6.69 (1.01; 54.36) | 0.055 |
| *Gastroenterology & Hepatology* | 1.26 (0.39; 5.66) | 0.721 |
| *General & Internal Medicine* | 2.25 (0.68; 10.20) | 0.224 |
| *General Clinical Medicine* | 3.40 (0.99; 15.83) | 0.074 |
| *Genetics & Heredity* | 1.33 (0.28; 7.33) | 0.722 |
| *Geriatrics* | 0.95 (0.15; 5.94) | 0.956 |
| *Gerontology* | 3.97 (0.63; 29.16) | 0.149 |
| *Health Policy & Services* | 4.17 (0.38; 49.72) | 0.230 |
| *History of Science, Technology & Medicine* | 1481285.82 (0.00; NA) | 0.965 |
| *Immunology* | 2.30 (0.72; 10.26) | 0.204 |
| *Legal & Forensic Medicine* | 3.11 (0.84; 15.24) | 0.114 |
| *Microbiology* | 2.76 (0.87; 12.27) | 0.120 |
| *Mycology & Parasitology* | 1.98 (0.40; 11.34) | 0.410 |
| *Neurology & Neurosurgery* | 1.63 (0.52; 7.16) | 0.452 |
| *Nuclear Medicine & Medical Imaging* | 1.15 (0.34; 5.27) | 0.834 |
| *Nutrition & Dietetics* | 3.13 (0.87; 14.97) | 0.105 |
| *Obstetrics & Reproductive Medicine* | 1.33 (0.41; 5.99) | 0.668 |
| *Oncology & Carcinogenesis* | 2.25 (0.72; 9.88) | 0.208 |
| *Ophthalmology & Optometry* | 0.55 (0.13; 2.77) | 0.419 |
| *Orthopedics* | 0.59 (0.14; 3.07) | 0.492 |
| *Otorhinolaryngology* | 0.65 (0.16; 3.27) | 0.566 |
| *Pathology* | 2.52 (0.62; 13.00) | 0.221 |
| *Pediatrics* | 2.02 (0.59; 9.33) | 0.304 |
| *Physiology* | 0.46 (0.02; 4.19) | 0.529 |
| *Psychiatry* | 1.55 (0.42; 7.51) | 0.535 |
| *Public Health* | 0.91 (0.10; 6.66) | 0.924 |
| *Rehabilitation* | 1.03 (0.16; 6.53) | 0.973 |
| *Respiratory System* | 1.11 (0.29; 5.42) | 0.888 |
| *Substance Abuse* | 0.00 (NA; NA) | 0.973 |
| *Surgery* | 0.30 (0.08; 1.43) | 0.088 |
| *Toxicology* | 7.66 (2.08; 37.57) | 0.005 |
| *Tropical Medicine* | 1.81 (0.27; 12.27) | 0.528 |
| *Urology & Nephrology* | 0.60 (0.17; 2.82) | 0.463 |
| *Virology* | 0.91 (0.17; 5.23) | 0.911 |
| **Zone** |  |  |
| *North of Italy* | 0.93 (0.77; 1.13) | 0.469 |
| *South of Italy* | 0.93 (0.73; 1.19) | 0.585 |

*n: number;NA:Not applicable*

**Data sharing statement**

**Data available**: Yes

**Data types:** Database is available from the original source: Ioannidis, John P.A. (2025), “August 2025 data-update for "Updated science-wide author databases of standardized citation indicators"”, Elsevier Data Repository, V8, doi: 10.17632/btchxktzyw.8.

Additional deidentified data (gender, age) is available from the authors

**How to access data**: Data can be requested from [alessandro.decassai@unipd.it](mailto:alessandro.decassai@unipd.it)

**When available**: With publication

**Who can access the data:** Anyone requesting the data.

**Types of analyses:** For any purpose.

**Mechanisms of data availability:** With investigator support.

A**ny additional restrictions**: None.
