# Supplementary material for: Gender disparities in Italian academic medicine: A cross-sectional study of clinicians in the 2024 stanford top 2% scientists database
Source: PLoS One. 2026 Apr 24;21(4):e0347836. doi: 10.1371/journal.pone.0347836 (PMC13108791; doi:10.1371/journal.pone.0347836)
Supplement: Attachment — Submitted filename: Reviewers#3 (2).docx [file pone.0347836.s005.docx]

Padova 01 April 2026

Dear Editor,

we have revised our manuscript according to your comments.

EDITOR COMMENT:

Q1:“Specifically, reviewer 2 asked "substantiating evidence for the cultural reference to naming conventions in Italy, normally cultural adduction is not done in scientific writing without empirical justification."

A1:we have modified the text in order to strength our methods as follows:

“To minimize misclassification bias, we restricted our analysis to Italian scientists, as Italian given names allow gender inference with high accuracy due to morphological regularities, although exceptions prevent deterministic classification. Moreover, the Italian National Institute of Statistics (ISTAT), the official governmental body responsible for collecting and analyzing demographic data in Italy, provides a publicly accessible database (<https://www.istat.it/dati/calcolatori/contanomi/>; last accessed 01 April 2026) listing Italian given names along with the corresponding percentages of individuals by gender.”

REVIEWERS COMMENTS

Reviewer #2:

R2: All Reviewer comments have been successfully addressed, no further changes appear needed except perhaps substantiating evidence for the cultural reference to naming conventions in Italy, normally cultural adduction is not done in scientific writing without empirical justification.

A2::we have modified the text in order to strength our methods as follows:

“To minimize misclassification bias, we restricted our analysis to Italian scientists, as Italian given names allow gender inference with high accuracy due to morphological regularities, although exceptions prevent deterministic classification. Moreover, the Italian National Institute of Statistics (ISTAT), the official governmental body responsible for collecting and analyzing demographic data in Italy, provides a publicly accessible database (<https://www.istat.it/dati/calcolatori/contanomi/>; last accessed 01 April 2026) listing Italian given names along with the corresponding percentages of individuals by gender.”

Reviewer #3

R3: Agradeço ao(s) autoro (es) a submissão da versão revisada do manuscrito e por considerar cuidadosamente os comentários levantados durante o processo de revisão.
O(s) autor(es) respondeu de forma satisfatória e construtiva os apontamentos na revisão inicial. As revisões aprimoraram a clareza e a qualidade geral do trabalho. Dessa forma. recomendo a aceitação do seu manuscrito em seu formato atual.

A3: We would like to thank the Reviewer for his help in proving aour manuscript
